# Impact of diabetes mellitus on mortality in patients with acute heart failure: a prospective cohort study

**DOI:** 10.1186/s12933-020-01026-3

**Published:** 2020-05-02

**Authors:** Min Gyu Kong, Se Yong Jang, Jieun Jang, Hyun-Jai Cho, Sangjun Lee, Sang Eun Lee, Kye Hun Kim, Byung-Su Yoo, Seok-Min Kang, Sang Hong Baek, Dong-Ju Choi, Eun-Seok Jeon, Jae-Joong Kim, Myeong-Chan Cho, Shung Chull Chae, Byung-Hee Oh, Soo Lim, Sue K. Park, Hae-Young Lee

**Affiliations:** 1 Department of Internal Medicine, Soon Chun Hyang University Hospital, Bucheon, South Korea; 2 Department of Internal Medicine, School of Medicine, Kyungpook National University, Daegu, South Korea; 3 Department of Preventive Medicine, Seoul National University College of Medicine, Seoul, South Korea; 4 Department of Biomedical Science, Seoul National University Graduate School, Seoul, South Korea; 5 Cancer Research Institute, Seoul National University, Seoul, South Korea; 6 Department of Internal Medicine, Seoul National University Hospital, Seoul, South Korea; 7 Department of Internal Medicine, Asan Medical Center, Seoul, South Korea; 8 Heart Research Center of Chonnam National University, Gwangju, South Korea; 9 Department of Internal Medicine, Yonsei University Wonju College of Medicine, Wonju, South Korea; 10 Department of Internal Medicine, Yonsei University College of Medicine, Seoul, South Korea; 11 Department of Internal Medicine, Catholic University of Korea, Seoul, South Korea; 12 Department of Internal Medicine, Seoul National University College of Medicine, Seoul National University Bundang Hospital, Seongnam, South Korea; 13 Department of Internal Medicine, Sungkyunkwan University School of Medicine, Seoul, South Korea; 14 Department of Internal Medicine, Chungbuk National University College of Medicine, Cheongju, South Korea; 15 Division of Cardiology, Department of Internal Medicine, Seoul National University College of Medicine, 101 Daehak-ro, Jongno-gu, Seoul, 03080 South Korea

**Keywords:** Diabetes mellitus, Acute heart failure, Left ventricular ejection fraction, Glycemic control

## Abstract

**Background:**

Although more than one-third of the patients with acute heart failure (AHF) have diabetes mellitus (DM), it is unclear if DM has an adverse impact on clinical outcomes. This study compared the outcomes in patients hospitalized for AHF stratified by DM and left ventricular ejection fraction (LVEF).

**Methods:**

The Korean Acute Heart Failure registry prospectively enrolled and followed 5625 patients from March 2011 to February 2019. The primary endpoints were in-hospital and overall all-cause mortality. We evaluated the impact of DM on these endpoints according to HF subtypes and glycemic control.

**Results:**

During a median follow-up of 3.5 years, there were 235 (4.4%) in-hospital mortalities and 2500 (46.3%) overall mortalities. DM was significantly associated with increased overall mortality after adjusting for potential confounders (adjusted hazard ratio [HR] 1.11, 95% confidence interval [CI] 1.03–1.22). In the subgroup analysis, DM was associated with higher a risk of overall mortality in heart failure with reduced ejection fraction (HFrEF) only (adjusted HR 1.14, 95% CI 1.02–1.27). Inadequate glycemic control (HbA1c ≥ 7.0% within 1 year after discharge) was significantly associated with a higher risk of overall mortality compared with adequate glycemic control (HbA1c < 7.0%) (44.0% vs. 36.8%, log-rank p = 0.016).

**Conclusions:**

DM is associated with a higher risk of overall mortality in AHF, especially HFrEF. Well-controlled diabetes (HbA1c < 7.0%) is associated with a lower risk of overall mortality compared to uncontrolled diabetes.

*Trial registration* ClinicalTrial.gov, NCT01389843. Registered July 6, 2011. https://clinicaltrials.gov/ct2/show/NCT01389843

## Background

Around 26 million people suffer from heart failure (HF) globally, and the prevalence is increasing with an increasing longevity, prevalence of risk factors, and improved survival in patients with cardiovascular diseases [1, 2]. In the United States, HF is the primary cause of hospitalization among patients aged > 65 years [3]. Hospitalization for HF is associated with a high mortality and rate of re-hospitalization [4, 5]. Around 75% patients with HF have ≥ 1 comorbidity, and these comorbidities make overall clinical outcomes worse [6]. In a recent meta-analysis, patients with diabetes mellitus (DM) were suggested to have a two-fold increase in the risk of HF [7]. DM is present in ~ 35% patients hospitalized with acute HF [8]. Multiple factors such as ischemia, hypertension, and extracellular fluid volume expansion are involved in the pathogenesis of HF in DM [9, 10]. While DM is associated with an increased cardiovascular morbidity and mortality in patients with chronic HF with reduced left ventricular ejection fraction (HFrEF) [11, 12], its independent impact on in-hospital and long-term outcomes after HF hospitalization is unclear. Data from some large registries and clinical trials suggest that DM is associated with worse in-hospital and post-discharge outcomes in patients with acute HF [13–18]. Other studies do not suggest a significant association of DM with mortality in patients hospitalized for HF after adjusting for confounding factors [19–22]. Thus, the independent association of DM with mortality in patients with HF remains unknown. It is also unclear if DM has similar adverse impact across HF subtypes such as HFrEF, HF with preserved ejection fraction (HFpEF), or HF with mid-range ejection fraction (HFmrEF).

We compared acute HF-associated in-hospital and overall all-cause mortality in patients with and without DM using the Korean Acute Heart Failure Registry (KorAHF) [23]. We also compared the outcomes in each HF subtype.

## Methods

### Study population

We evaluated the patients with acute HF enrolled in the KorAHF registry (ClinicalTrial.gov identifier, NCT01389843) [23]. Briefly, the KorAHF registry is a prospective multicenter cohort study of 5625 patients admitted for acute heart failure (AHF) in 10 tertiary university hospitals between March 2011 and February 2014 who have been followed for > 5 years until February 2019. Patients who had signs or symptoms of HF and met ≥ 1 of the following criteria were enrolled in this registry: (1) lung congestion or (2) objective evidence of left ventricular (LV) systolic dysfunction or (3) structural heart disease.

**Fig. 1:**
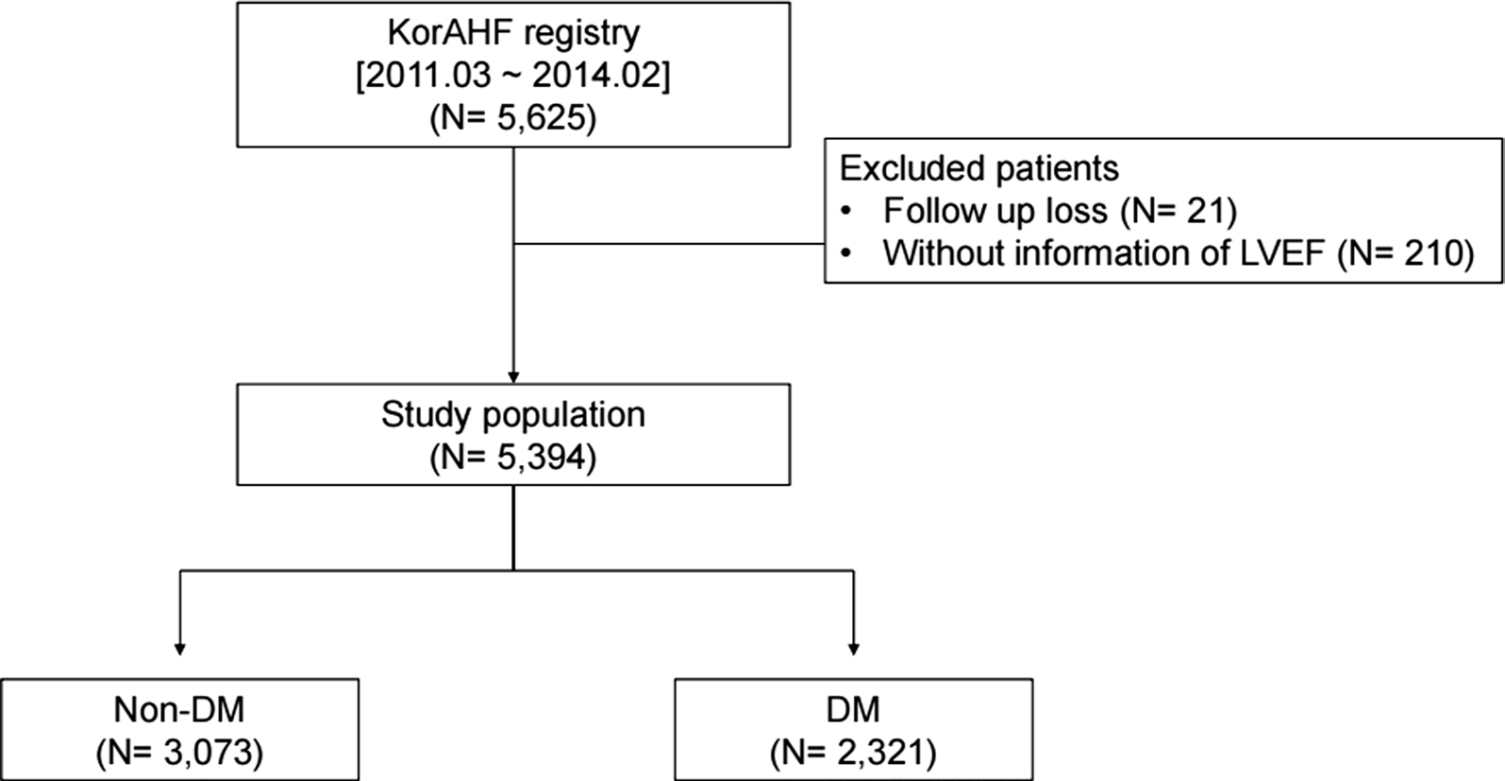
Flow chart of the study. KorAHF registry, Korean Acute Heart Failure registry

We excluded 210 patients where there was no information on LV ejection fraction (LVEF) and 21 patients who were lost to follow-up. Finally, 5394 patients with AHF and known DM status and LVEF were enrolled for analyses (Fig. 1).

### Data collection and outcome definition

Data were collected at each hospital and entered into a web-based Clinical Research and Trial (iCReaT) system case-report form of the Korea National Institute of Health. Detailed information was collected at the time of admission, and follow-up data were collected from the patients by the attending physician at 30 days and 3, 6, 12, 24, 36, 48, and 60 months after discharge. Data on patient demographics, medical history, physical signs, laboratory test results, electrocardiography, echocardiography, medications, and outcomes were collected. The mortality data for patients lost to follow-up was collected from the National Insurance data or National Death Records.

### Definition of DM and glycemic control

DM was defined as self-reported, history of anti-hyperglycemic agent use, or newly diagnosed during hospitalization [17]. Newly diagnosed DM was defined as a glycated hemoglobin (HbA1c) ≥ 6.5% when measured after a random glucose level ≥ 200 mg/dl at enrollment. We additionally classified DM patients based on HbA1c levels measured at the follow-up visit ≤ 1 year from discharge. We defined well-controlled and uncontrolled DM by an HbA1c < 7.0% and ≥ 7.0% at the follow-up visit, respectively. According to LVEF, we categorized patients with AHF into 3 groups: LVEF < 40% (HFrEF), 40% ≤ LVEF < 50% (HFmrEF), and LVEF ≥ 50% (HFpEF).

### Statistical analysis

Baseline characteristics as per DM status were compared using the χ^2^ test for categorical variables and the unpaired Student’s *t*-test for continuous variables. Kaplan–Meier survival curves as per DM status were compared using the log-rank test. We used the multivariable Cox proportional hazard regression model to evaluate the association between DM and mortality in patients with AHF. Potential confounders which were different at baseline in patients with and without DM, or were considered clinically significant including age, sex, body mass index (BMI), etiology of HF (ischemic or non-ischemic), prior admission for HF, use of parenteral inotropic agents, serum creatinine concentration (< 2.0 or ≥ 2.0 mg/dL), elevated brain natriuretic peptides (BNP) (≥ 500 pg/mL) or N-terminal pro-brain natriuretic peptides (NT-proBNP) (≥ 1000 pg/mL), New York Heart Association (NYHA) class (III–IV or I–II) on admission, and smoking status (current or ex-smoker vs. never-smoker) were adjusted for in the multivariable model. An interaction between DM and potential confounders was assessed by adding interaction terms in the Cox proportional hazard regression model. All p-values were two-sided, and p-values < 0.05 were considered statistically significant. Statistical analyses were performed using SAS software version 9.4 (SAS Institute Inc., Cary, NC, USA) and R version 3.6.0 with packages (“survival”, and “survminer”).

## Results

### Baseline characteristics

In the study population, 2321 patients with AHF had DM (43.0%) (Table 1). Patients with DM had a higher prevalence of risk factors like old age, obesity, hypertension, ischemic heart disease, chronic kidney disease, and cerebrovascular disease. Patients with DM had a higher proportion of patients with a BNP ≥ 500 pg/mL or NT-proBNP ≥ 1000 pg/mL, NYHA class III-IV on admission, acute pulmonary edema on chest X-ray, a higher level of systolic blood pressure, C-reactive protein, serum potassium and creatinine concentration, and lower serum sodium concentration and LVEF compared to those without DM. Besides, patients with DM were more likely to be on parenteral diuretics, inotropic agents, and vasodilators. However, aldosterone antagonists were prescribed less frequently in patients with DM.

**Table 1:** Baseline clinical characteristics according to diabetes mellitus (DM)

Variables	All patients (N = 5394)	Non-DM (N = 3073)	DM (N = 2321)	*P*-value
Age	68.5 ± 14.5	67.6 ± 15.9	69.6 ± 12.3	< 0.001
Body mass index (kg/m^2^)	23.0 ± 3.9	23.0 ± 3.9	23.7 ± 3.8	< 0.001
Male, N (%)	2872 (53.2)	1596 (51.9)	1277 (55.0)	0.023
Current smoker, N (%)	961 (17.8)	546 (17.8)	415 (17.9)	0.086
Risk factors, N (%)				
Hypertension	3183 (59.0)	1554 (50.6)	1629 (70.2)	< 0.001
Ischemic heart disease	1501 (27.8)	636 (20.7)	865 (37.2)	< 0.001
Atrial fibrillation	1523 (28.2)	921 (30.0)	602 (25.9)	0.001
Chronic lung disease	608 (11.3)	350 (11.4)	258 (11.1)	0.492
Chronic kidney disease	756 (14.0)	277 (9.0)	479 (20.6)	< 0.001
Cerebrovascular disease	807 (15.0)	405 (13.2)	402 (17.3)	< 0.001
Previous heart failure	2539 (47.1)	1380 (44.9)	1159 (49.9)	< 0.001
Physical and laboratory findings				
SBP, mmHg	131.4 ± 30.1	130.4 ± 29.4	132.8 ± 30.9	0.003
DBP, mmHg	78.7 ± 18.7	79.2 ± 18.8	78.1 ± 18.6	0.028
Heart rate, beats/min	92.8 ± 25.9	92.5 ± 26.4	93.1 ± 25.2	0.379
Glucose, mg/dL	155.3 ± 76.7	129.6 ± 47.8	189.1 ± 94.1	< 0.001
Total cholesterol, mg/dL	151.8 ± 43.2	153.9 ± 42.2	149.2 ± 44.4	< 0.001
BNP ≥ 500 pg/mL or NT-proBNP ≥ 1000 pg/mL	4047 (75.0)	2267 (73.8)	1780 (76.7)	0.014
CRP, mg/dL	2.4 ± 4.3	2.1 ± 3.5	2.9 ± 5.0	< 0.001
hsCRP, mg/dL	2.3 ± 4.2	2.0 ± 3.8	2.6 ± 4.6	< 0.001
Sodium, mmol/L	137.5 ± 4.8	138.0 ± 4.6	136.8 ± 5.0	< 0.001
Potassium, mmol/L	4.4 ± 0.7	4.3 ± 0.6	4.5 ± 0.8	< 0.001
BUN, mg/dL	26.1 ± 16.3	23.7 ± 14.3	29.2 ± 18.3	< 0.001
Creatinine, mg/dL	1.5 ± 1.5	1.3 ± 1.3	1.7 ± 1.6	< 0.001
NYHA class III-IV, N (%)	4582 (84.9)	2558 (83.2)	2024 (87.2)	< 0.001
Acute pulmonary edema on chest X-ray, N (%)	1039 (19.3)	502 (16.3)	537 (23.1)	< 0.001
Echocardiographic findings				
LVEDD, mm	57.4 ± 10.1	57.5 ± 10.6	57.4 ± 9.3	0.863
LVESD, mm	45.2 ± 12.3	45.1 ± 12.8	45.4 ± 11.7	0.302
LVEF (%)	37.8 ± 15.6	38.5 ± 15.9	36.7 ± 15.0	< 0.001
LA volume index, mL/m^2^	63.8 ± 42.1	66.7 ± 41.9	59.6 ± 42.0	< 0.001
E′, cm/s	5.0 ± 2.3	5.2 ± 2.1	4.8 ± 2.5	< 0.001
S′, cm/s	5.1 ± 2.0	5.1 ± 2.1	5.0 ± 1.9	0.026
E/E′	21.2 ± 11.5	20.1 ± 10.8	22.7 ± 12.2	< 0.001
RVSP	43.9 ± 15.1	43.2 ± 14.9	44.9 ± 15.4	< 0.001
Management, N (%)				
Parenteral diuretics	4062 (75.3)	2222 (72.3)	1840 (79.3)	< 0.001
Parenteral inotropics	1672 (31.0)	760 (24.7)	912 (39.3)	< 0.001
Parenteral vasodilators	2231 (41.4)	1105 (36.0)	1126 (48.5)	< 0.001
ACEIs/ARBs at admission	3383 (62.7)	1977 (64.3)	1406 (60.6)	0.001
ACEIs/ARBs at discharge	3601 (66.8)	2117 (68.9)	1484 (63.9)	< 0.001
Beta-blockers at admission	2054 (38.1)	1183 (38.5)	871 (37.5)	0.001
Beta-blockers at discharge	2725 (50.5)	1533 (49.9)	1192 (51.4)	0.285
AAs at admission	2206 (40.9)	1379 (44.9)	827 (35.6)	< 0.001
AAs at discharge	2443 (45.3)	1472 (47.9)	971 (41.8)	< 0.001
Warfarin at discharge	1531 (28.4)	965 (31.4)	566 (24.4)	< 0.001
Heart transplantation	69 (1.3)	13 (0.4)	56 (2.4)	< 0.001

Values are presented as mean ± standard deviation, or n (%)

*DM* diabetes mellitus, *SBP* systolic blood pressure, *DBP* diastolic blood pressure, *BNP* brain natriuretic peptides, *NT-proBNP* N-terminal pro-brain natriuretic peptides, *hsCRP* high sensitivity C-reactive protein, *CRP* C-reactive protein, *BUN* blood urea nitrogen, *LVEDD* left ventricular end-diastolic dimension, *LVEDV* left ventricular end-diastolic volume, *LVEF* left ventricular ejection fraction, *LA* left atrium, *RVSP* right ventricular systolic pressure, *ACEIs* angiotensin converting enzyme inhibitors, *ARBs* angiotensin receptor blockers, *AAs* aldosterone antagonists

All patients underwent echocardiography during their index admission (Table 1). There were no significant differences in the LV end-diastolic dimension (LVEDD) and LV end-systolic dimension (LVESD) between the two groups. However, there was a significant difference in the LVEF (38.5 ± 15.9% vs. 36.7 ± 15.0%, p < 0.001). Furthermore, LV diastolic function parameters such as E/e′ (20.1 ± 10.8 vs. 22.7 ± 12.2, p < 0.001) and right ventricular (RV) systolic pressure (43.2 ± 14.9 mmHg vs. 44.9 ± 15.4 mmHg, p < 0.001) were worse in patients with DM. Conversely, patients without DM had a larger LA volume index (66.7 ± 41.9 mL/m^2^ vs. 59.6 ± 42.0 mL/m^2^, p < 0.001).

### In-hospital and overall mortality as per DM status

**Fig. 2:**
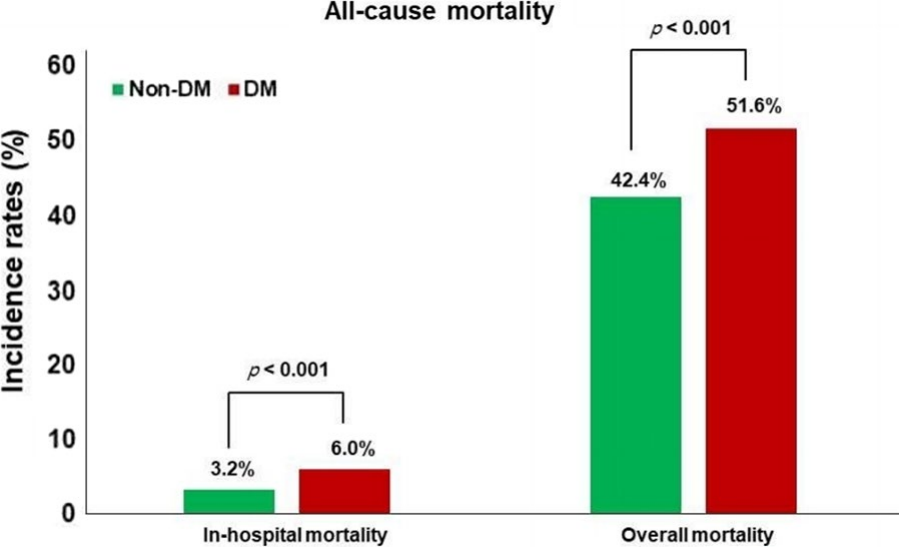
Comparison of in-hospital and overall all-cause mortality as per DM status

During a median follow-up of 3.5 years, there were 235 (4.4%) deaths during the index hospitalization, and 2500 (46.3%) deaths during the overall follow-up period. Patients with DM had a higher incidence of in-hospital mortality and overall mortality compared to patients without DM (Fig. 2). After adjusting for potential confounders including age, sex, BMI, etiology of heart failure (ischemic vs. non-ischemic), prior admission for HF, parenteral inotropic use, serum creatinine concentration, elevated BNP/NT-proBNP, NYHA class III-IV on admission, and smoking status, DM was still independently associated with overall mortality (adjusted hazard rate [HR] 1.11, 95% confidence interval [CI] 1.03–1.22).

### Independent predictors of in-hospital and overall mortality

Results of multivariable Cox proportional hazard regression for in-hospital and overall all-cause mortality are reported in Table 2. DM was not independently associated with an increased in-hospital mortality (HR 0.81, 95% CI 0.61–1.07, p = 0.137). Use of parenteral inotropes, age, ischemic etiology, and a higher serum creatinine concentration also independently predicted in-hospital mortality.

**Table 2:** Independent predictors of in-hospital and overall mortality on multivariable Cox proportional hazard regression model

Variables	Adjusted HR^a^	*P* value
In-hospital mortality		
DM	0.81 (0.61–1.07)	0.137
Age (years)	1.03 (1.02–1.04)	< 0.001
Ischemic cause (vs non-ischemic cause)	1.41 (1.07–1.86)	0.016
Parenteral inotropics usage	5.14 (3.43–7.68)	< 0.001
Serum creatinine ≥ 2.0 (vs < 2.0 mg/dL)	1.54 (1.15–2.07)	0.015
Overall mortality		
DM	1.11 (1.03–1.22)	0.013
Age (years)	1.04 (1.04–1.05)	< 0.001
Sex (male)	1.26 (1.14–1.38)	< 0.001
Body mass index (kg/m^2^)		
Underweight vs. Normal	1.66 (1.47–1.88)	< 0.001
Overweight or obese vs. Normal	0.80 (0.73–0.89)	< 0.001
Ischemic cause (vs non-ischemic cause)	1.17 (1.07–1.27)	< 0.001
Prior admission history due to HF	1.51 (1.39–1.64)	< 0.001
Parenteral inotropics usage	1.41 (1.30–1.55)	< 0.001
Serum creatinine ≥ 2.0 (vs < 2.0 mg/dL)	1.63 (1.50–1.83)	< 0.001
Higher BNP (≥ 500), or NT-proBNP (≥ 1000) during index hospitalization	1.32 (1.22–1.49)	< 0.001
NYHA class III–IV on admission	1.35 (1.22–1.49)	< 0.001

^a^Adjusted for age, sex, body mass index, etiology of heart failure (ischemic vs. non-ischemic), prior admission history due to HF, parenteral inotropics usage, creatinine concentration (< 2.0 vs. ≥ 2.0 mg/dL), elevated BNP (≥ 500) or NT-proBNP (≥ 1000), NYHA class (III-IV or I-II) on admission, and smoking status (current or ex-smoker vs. never-smoker)

DM was an independent predictor for overall mortality (HR 1.11, 95% CI 1.03–1.22, p = 0.013). Other variables, such as old age, male sex, higher BMI, ischemic etiology, acute decompensated HF, use of parenteral inotropes, high concentrations of serum creatinine and BNP/NT-proBNP during index hospitalization, and NYHA class III-IV on admission also independently predicted higher overall mortality.

### In-hospital and overall mortality according to DM in subgroup by LVEF

Patients with DM had a higher in-hospital mortality rate vs. patients without DM in all LVEF subgroups (HFrEF 7.1% vs. 3.4%, HFmrEF 4.3% vs. 3.2%, HFpEF 3.8% vs. 2.7%). However, there was no significant association of DM with higher in-hospital mortality rate after adjusting for potential confounders (HFrEF, adjusted HR 0.96, 95% CI 0.68–1.35, HFmrEF, adjusted HR 0.71, 95% CI 0.33–1.53, HFpEF, adjusted HR 0.79, 95% CI 0.41–1.51) (Table 3).

**Table 3:** In-hospital and overall mortality according to DM in 3 subtypes of HF

Diabetes mellitus (DM)	Unadjusted HR (95% CI)	Adjusted HR (95% CI)^1^
In-hospital mortality		
LVEF < 40%		
Non-DM	1.00	1.00
DM	1.28 (0.92–1.77)	0.96 (0.68–1.35)
40% ≤ LVEF < 50%		
Non-DM	1.00	1.00
DM	0.83 (0.41–1.68)	0.71 (0.33–1.53)
LVEF ≥ 50%		
Non-DM	1.00	1.00
DM	0.94 (0.50–1.77)	0.79 (0.41–1.51)
Overall mortality		
LVEF < 40%		
Non-DM	1.00	1.00
DM	1.48 (1.33–1.64)	1.14 (1.02–1.27)
40% ≤ LVEF < 50%		
Non-DM	1.00	1.00
DM	1.19 (0.98–1.44)	0.99 (0.80–1.22)
LVEF ≥ 50%		
Non-DM	1.00	1.00
DM	1.15 (0.98–1.35)	1.13 (0.96–1.34)

Adjusted for age, sex, body mass index, etiology of heart failure (ischemic vs. non-ischemic), prior admission history due to HF, parenteral inotropics usage, creatinine concentration (< 2.0 vs. ≥ 2.0 mg/dL), elevated BNP (≥ 500) or NTproBNP (≥ 1000), NYHA class (III–IV or I–II) on admission, and smoking status (current or ex-smoker vs. never-smoker)

**Fig. 3:**
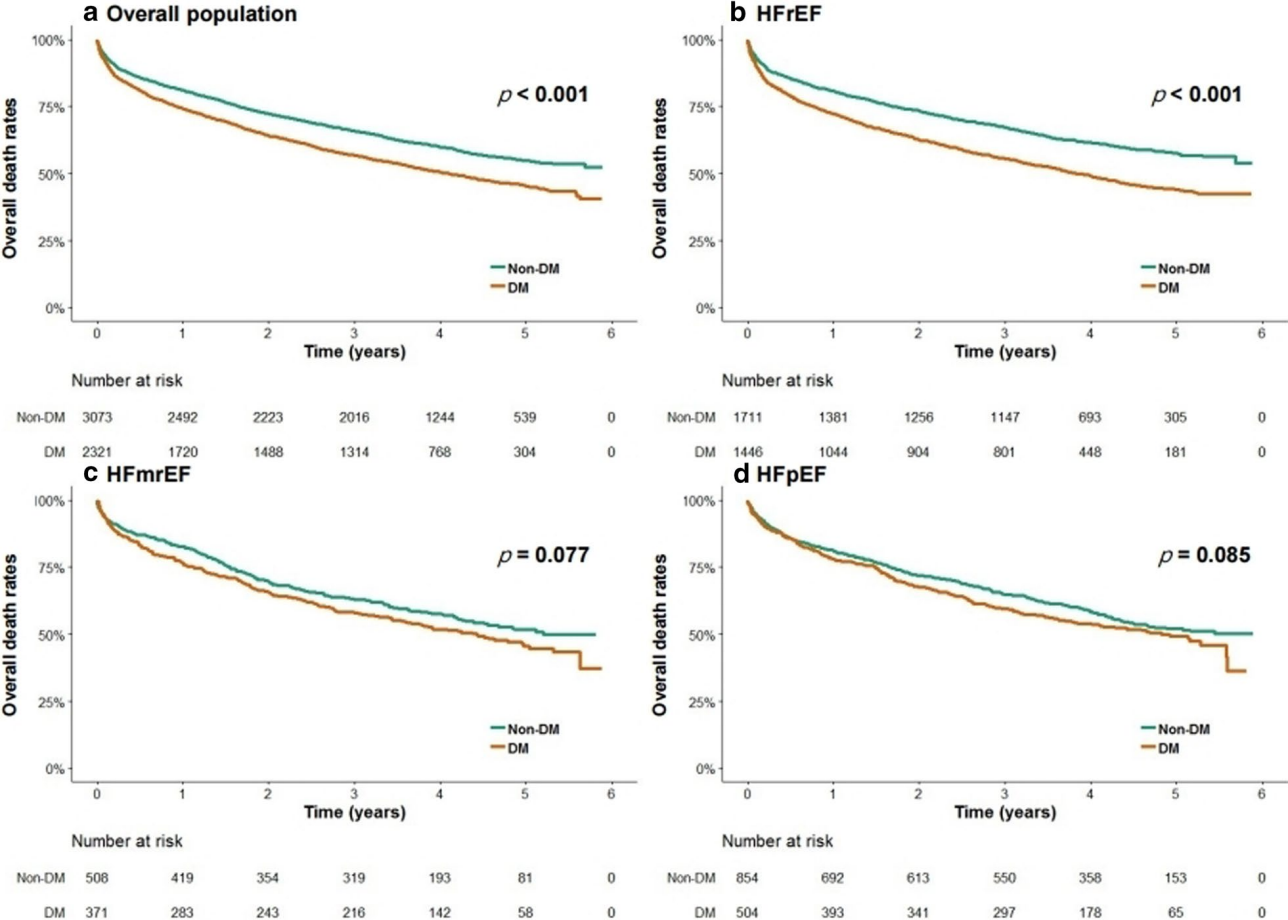
Kaplan–Meier curves of all-cause mortality according to DM in subgroup by LVEF

DM had differential impact on overall mortality as per the HF subtype. In HFrEF, DM was significantly associated with an increased risk of overall mortality after adjusting for potential confounders (adjusted HR 1.14, 95% CI 1.02–1.27). However, DM was not significantly associated with overall mortality in patients with HFmrEF (adjusted HR 0.99, 95% CI 0.80–1.22) and HFpEF (adjusted HR 1.13, 95% CI 0.96–1.34) (Table 3). The Kaplan–Meier analysis also revealed significantly worse overall mortality in patients with HFrEF and DM vs. HFrEF and no DM (40.2% vs. 52.7%, log-rank p < 0.001) (Fig. 3).

### Overall mortality as per the prespecified subgroup and glycemic control

**Fig. 4:**
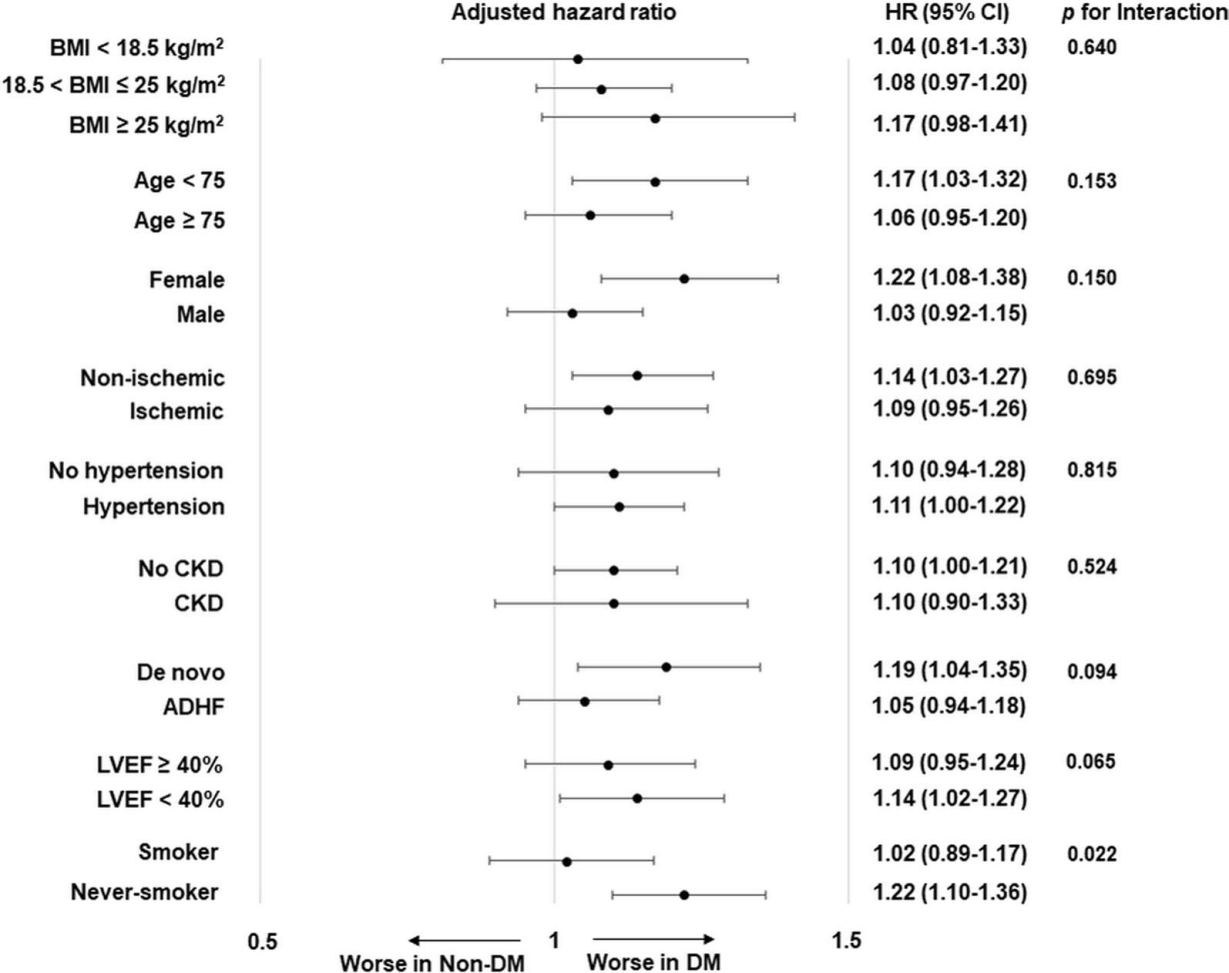
Overall all-cause mortality as per the prespecified subgroup

Figure 4 shows the association between DM and overall mortality in a stratified group as per the potential confounders, including age, sex, ischemic etiology, hypertension, chronic kidney disease, de novo HF, LVEF < 40%, and smoking status. The impact of DM on overall mortality was generally consistent across stratified subgroups (p-interaction ≥ 0.05). However, there was a significant difference in the impact of DM on overall mortality between smoker (current or ex-smoker) and never-smoker (p for interaction = 0.022).

**Fig. 5:**
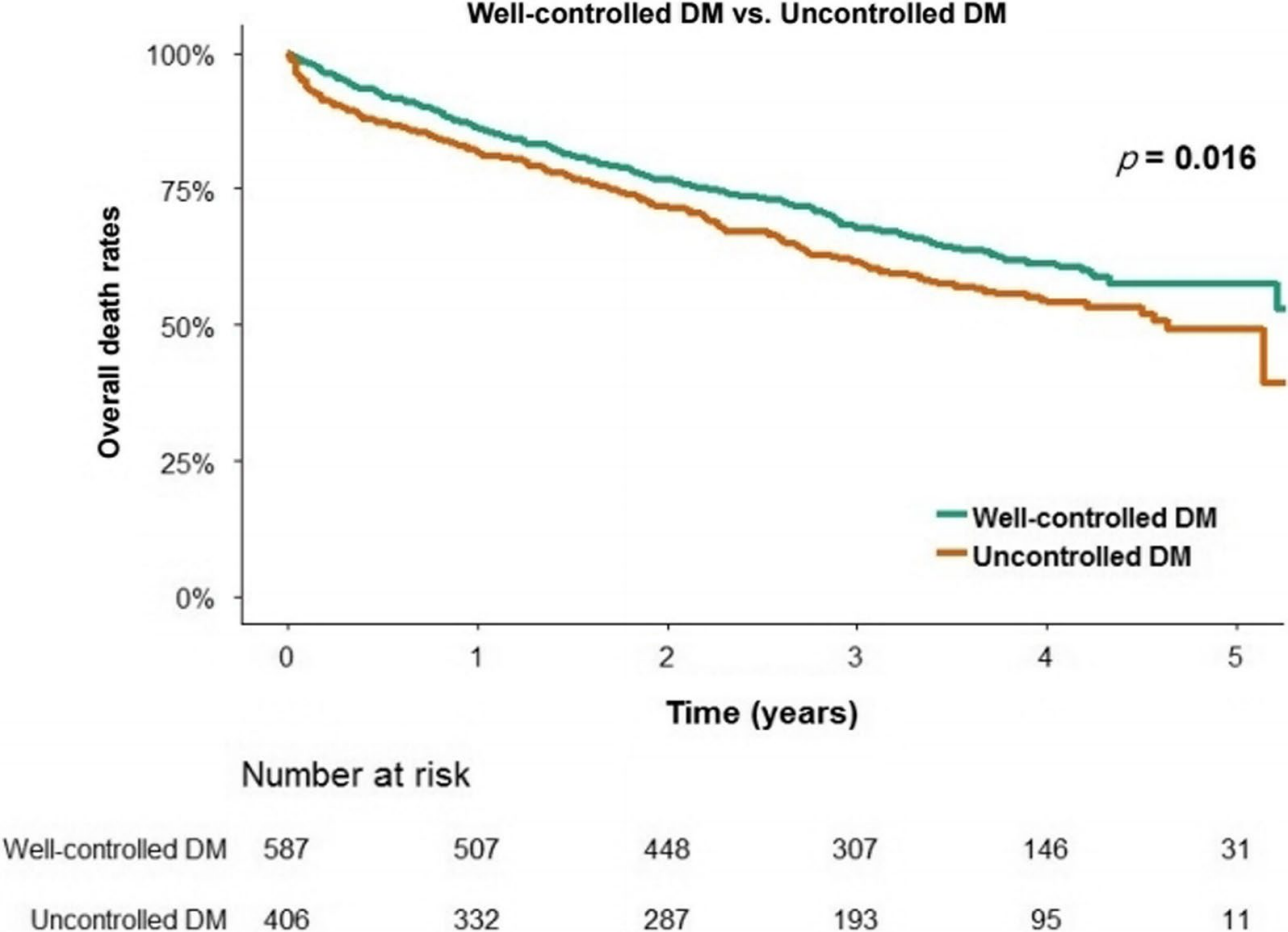
Overall all-cause mortality as per glycemic control after discharge in patients with DM. *Well-controlled DM as an HbA1c < 7.0% at a follow-up visit within 1 year after discharge; Uncontrolled DM as an HbA1c ≥ 7.0%

Figure 5 shows that patients with uncontrolled DM (HbA1c ≥ 7.0%) had significantly higher overall mortality compared to patients with well-controlled DM (HbA1c < 7.0%) by Kaplan–Meier analysis (44.0% vs. 36.8%, log-rank p = 0.016).

## Discussion

The main findings of our study are as follows: (1) patients with AHF and DM have a significantly higher in-hospital and overall mortality vs. patients with AHF and no DM; (2) DM was significantly associated with a higher overall mortality even after adjusting for potential confounding factors including age, sex, BMI, HF etiology, renal function, and HF severity; (3) DM had a significant association with higher overall mortality in HFrEF, but not HFmrEF and HFpEF; (4) patients with poor glycemic control after discharge (HbA1c ≥ 7.0%) had a higher overall mortality vs. patients with adequate glycemic control (HbA1c < 7.0%).

Previous studies in HF have compared the clinical characteristics and outcomes in patients with and without DM. However, there are few reports comparing clinical outcomes stratified by DM in HFpEF [12, 24, 25]. Moreover, there is no data from a large registry or clinical trials in patients with HFmrEF. The CHARM program demonstrated that DM was significantly associated with a higher mortality and morbidity in HFrEF and HFpEF [11]. Another large-scale study from the I-PRESERVE trial (Irbesartan in Heart Failure with Preserved Ejection Fraction) showed that patients with DM had more significant structural and functional echocardiographic abnormalities and worse clinical outcomes compared to patients without DM in HFpEF [24]. A recent prospective HFpEF study showed a significant association of DM with long-term mortality in women, but not in men [25]. Similar to HFrEF, these studies demonstrate a significant associations of DM with higher mortality in HFpEF. The mechanisms for poor prognosis of HF with DM are unclear.

Some of these mechanisms are: (1) DM causes microangiopathy, myocardial fibrosis, and autonomic neuropathy and these lead to diabetic cardiomyopathy [26]; (2) hyperglycemia leads to lipid accumulation in the heart, and this can cause cellular damage by lipotoxicity [27]; (3) lipid accumulation, collagen deposition and fibrosis, and hyperinsulinemia due to insulin resistance increases risk of hypertrophy of the heart [28, 29]; (4) DM may promote extracellular matrix expansion which is associated with a higher mortality in HF [30]; (5) impaired branched-chain amino acids catabolism and insulin signaling are associated with HF [31]; (6) distinct pathways related to inflammation, protein phosphorylation, and neutrophil degranulation are associated with DM in HF [32].

Why DM was not associated with an increased mortality in HFpEF and HFmrEF is unclear. The LVEF cutoff to classify HF in previous studies was different from the current updated guidelines for the diagnosis and treatment of HF that are accepted and used in clinical practice [33]. The CHARM program did not provide detailed echocardiographic data. The I-PRESERVE trial used an LVEF cutoff of 45%, and echocardiographic data were shown for < 20% of the whole study population. Our results require cautious interpretation. In general, patients with DM had a higher overall mortality, but this association was not statistically significant in HFpEF and HFmrEF after adjusting for risk factors such as old age, ischemic etiology, and severity of initial presentation.

Patients with HFmrEF have similar clinical characteristics as patients with HFpEF [34–37]. Recent studies demonstrate that mortality rates in HFmrEF are similar to those in HFpEF [35–38]. Although there are no studies on the association of DM with mortality in patients with HFmrEF, our study shows that this association is different from HFrEF and HFpEF. If HFmrEF is a distinct clinical syndrome or if these patients are in-transition between HFrEF and HFpEF is unknown [39]. Since there were limited patients with HFmrEF in our study, this association needs to be further explored.

Our study has important implications. First, we analyzed one of the largest prospective cohorts comparing the characteristics and clinical outcomes in patients with AHF, with and without DM. Second, our study analyzed baseline echocardiographic findings in all patients, which is unique and challenging to obtain in large HF registries. Third, we evaluated both in-hospital and overall all-cause mortality. This helped estimate both short and long-term impact of DM on mortality in patients with AHF. Fourth, we compared mortality in 3 subtypes of HF based on LVEF. To our best knowledge, this is the first study to evaluate the association of DM with mortality in HFrEF, HFmrEF, and HFpEF. Since the characteristics and prognosis of patients with HFpEF and HFmrEF are unknown, these results may help understand the clinical implications of HFpEF and HFmrEF. Lastly, we also demonstrate that an adequate glycemic control during follow-up was associated with an improved long-term prognosis in patients with AHF and DM.

### Limitations

There are several limitations of our study. First, this is an observational study. To evaluate the effect of glycemic control, it has intrinsic limitations of non-randomized comparisons such as the different distribution of other clinical risk factors and the possibility of unmeasured confounding factors. Second, our endpoint was only all-cause mortality. Detailed clinical outcomes such as cardiovascular death and re-hospitalization for HF may help better understand the impact of DM on outcomes in AHF. Third, many recent studies have evaluated the cardiovascular safety of anti-diabetic medications. While dipeptidyl peptidase 4 (DPP-4) inhibitors had a neutral effect, sodium-glucose co-transporter 2 (SGLT2) inhibitors were most favorable among all classes of anti-diabetic medications for reducing the risk of HF [40, 41]. However, SGLT2 inhibitors were not prescribed during the enrollment period of our registry. Therefore, our study could not evaluate their effect on HF.

## Conclusions

Our study, using large registry data with echocardiographic information from all participants, shows that DM is significantly associated with an increased risk of overall mortality in AHF, especially HFrEF. Well-controlled diabetes (HbA1c < 7.0%) was associated with a lower risk of overall mortality compared with uncontrolled diabetes (HbA1c ≥ 7.0%) in patients with AHF and DM.

## Data Availability

The data of this study may be available on reasonable request to the Korean Acute Heart Failure (KorAHF) Registry.

## Abbreviations

AHF: Acute heart failure
CI: Confidence interval
DM: Diabetes mellitus
HF: Heart failure
HFrEF: Heart failure with reduced ejection fraction
HFpEF: Heart failure with preserved ejection fraction
HFmrEF: Heart failure with mid-range ejection fraction
HR: Hazard ratio
LVEF: Left ventricular ejection fraction
NYHA: New York Heart Association
